# Developing novel antifungals: lessons from G protein-coupled receptors

**DOI:** 10.1016/j.tips.2022.12.002

**Published:** 2023-03-01

**Authors:** Vaithish Velazhahan, Bethany L. McCann, Elaine Bignell, Christopher G. Tate

**Affiliations:** 1 Medical Research Council (MRC) Laboratory of Molecular Biology, Francis Crick Avenue, Cambridge CB2 0QH, UK; 2 MRC Centre for Medical Mycology, Stocker Road, University of Exeter, Exeter EX4 4QD, UK

## Abstract

Up to 1.5 million people die yearly from fungal disease, but the repertoire of antifungal drug classes is minimal and the incidence of drug resistance is rising rapidly. This dilemma was recently declared by the World Health Organization as a global health emergency, but the discovery of new antifungal drug classes remains excruciatingly slow. This process could be accelerated by focusing on novel targets, such as G protein-coupled receptor (GPCR)-like proteins, that have a high likelihood of being druggable and have well-defined biology and roles in disease. We discuss recent successes in understanding the biology of virulence and in structure determination of yeast GPCRs, and highlight new approaches that might pay significant dividends in the urgent search for novel antifungal drugs.

## Fungal diseases and drug resistance, an ever-growing problem

Deaths per annum from fungal diseases exceed those caused by tuberculosis or malaria [1–3]. Most occur despite antifungal treatment, and mortality rates approach that of Ebola virus disease. Because rates of antifungal resistance are rapidly increasing, urgent action to address this threat is needed [4,5]. However, the slow rate at which novel antifungal drugs have been discovered and licensed (one new class in the current century [6,7]) has been outpaced by the capacity of fungal pathogens to acquire resistance in patients or in the environment [8]. Therefore, the characterization of new drug targets to overcome the continued threat of antifungal resistance is urgently required, particularly in light of an ever-expanding cohort of at-risk patients suffering from severe respiratory infections such as chronic obstructive pulmonary disease (COPD) and coronavirus disease 2019 (COVID-19). Current antifungal drugs target only a very limited number of proteins, and none of these are **G protein-coupled receptors (GPCRs)** (see Glossary) or ion channels, whereas in humans 53% of all clinical drugs target these two protein families. A major difference between human and fungal cells is the presence of the cell wall in fungi, which could in theory impede drug access to membrane proteins in the plasma membrane. However, the yeast secretome contains enzymes up to 200 kDa in molecular weight [9], and the size of pores in the cell wall would therefore not impede drug diffusion.

Recent developments in structural biology [10,11], the so-called ‘resolution revolution’ in **electron cryo-microscopy (cryo-EM**), have opened up new opportunities for drug discovery by facilitating the rapid determination of **protein structures** and subsequent **structure-based drug discovery (SBDD)**. The rapidity with which new structures of human GPCRs are being determined is now breathtaking [12], and could not have been imagined even 5 years ago. Once the structure of a protein has been determined, billions of small-molecule compounds can be screened computationally to rapidly identify novel inhibitors/activators of proteins [13,14]. Thus, if we could determine structures of membrane proteins from pathogenic fungi, then the tools are available to make rapid progress in identifying molecules that could activate/inhibit them.

Here we briefly review current modes of action of antifungal drugs and the current state of drug discovery to human GPCRs. We then discuss how the characterization of new membrane protein targets in pathogenic fungi could be rapidly leveraged using structural biology and computational approaches to identify new small molecules that have the potential to be developed into antifungal drugs.

## Antifungal modes of action: old and new

**Figure 1:**
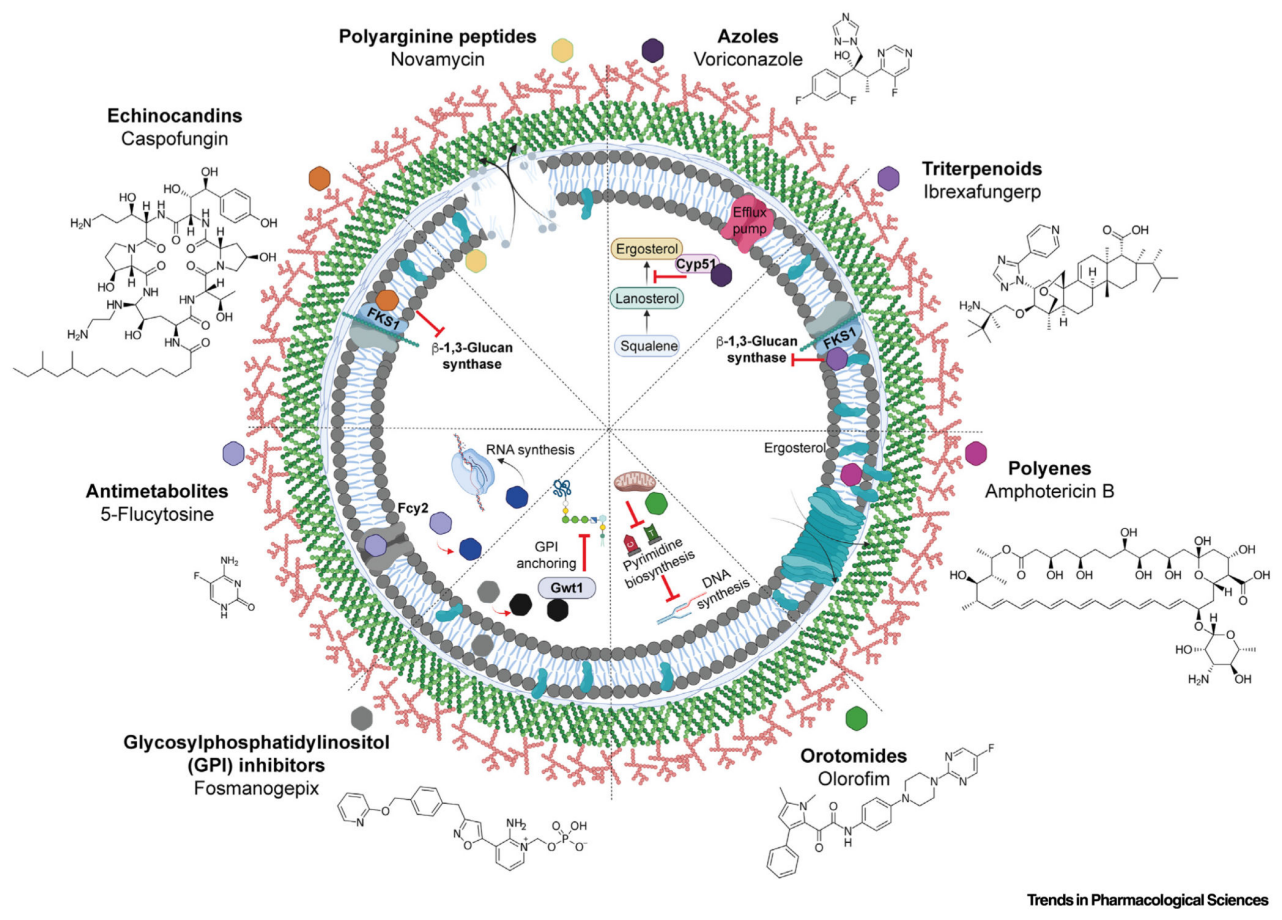
Mechanism of action of antifungal agents. Azoles (inhibit ergosterol synthesis), echinocandins (inhibit β-1,3 glucan synthesis), and polyenes (generate membrane pores) are the most commonly used antifungals in the treatment of serious fungal infections, all of which target the cell periphery to disrupt cellular integrity. The antimetabolite 5-flucytosine (5FC), which is most often used in combination with amphotericin B in the treatment of candidemia or cryptococcosis, inhibits RNA synthesis. Several promising antifungals are under development and in clinical trials. Ibrexafungerp inhibits β-1,3 glucan synthesis (like caspofungin) through interaction with the catalytic FKS1 subunit of β-1,3 glucan synthase. Olorofim is a first-in-class orotomide that dysregulates DNA (pyrimidine) synthesis through inhibition of dihydroorotate dehydrogenase. Manogepix (the active derivative of the prodrug fosmanogepix) disrupts glycosylphosphatidylinositol (GPI) anchoring at the cell periphery via inhibition of Gwt1. Novamycin (NP339) partitions into the plasma membrane and results in the formation of membrane pores. Image created using BioRender.

For the past 70 years only four classes of antifungal drug, mostly used as monotherapy, have provided the basis of all systemic antifungal therapy. Repeatedly, resistance to new antifungal drugs has occurred rapidly after their introduction into the clinic or following changes in prescribing practice. Although multiple new agents are becoming available, their resistance liabilities remain unknown. Most currently available antifungal drugs exert their effects by disrupting essential structural features of the cellular periphery, including the plasma membrane and cell wall. Figure 1 shows a schematic representation of the modes of action, and known resistance mechanisms, of licensed antifungal drugs and newer agents that are currently undergoing clinical trials. The azole class of antifungals serves as a frontline of defense against many different types of invasive fungal infections (e.g., [15]). Azoles inhibit the production of the essential plasma membrane sterol ergosterol via inhibition of 14α-lanosterol demethylase, encoded in yeasts by *erg11*, or *cyp51A* in filamentous fungi [16–18]. Resistance to azoles is rapidly increasing and is caused by several different mechanisms, often occurring simultaneously, including mutations in *erg11* or *cyp51* or their promoter regions, or upregulation of efflux pumps [6,19,20]. Extensive use of azoles in agriculture is also thought to contribute to the increased frequency of drugresistant strains isolated in the clinic [8]. **Echinocandins** and the first antifungal drug of the **triterpenoid** class, ibrexafungerp, target the FK506 sensitivity subunit **(FKS1 subunit**) of β-1,3-glucan synthase [21], which catalyzes the production of a major and essential cell-wall component β-1,3-glucan. Resistance to echinocandins is primarily associated with mutations in FKS1 [22–24]. Polyenes such as amphotericin B and its liposomal formulation AmBisome interact with plasma membrane-associated ergosterol resulting in formation of membrane pores and reduced membrane integrity that has rapid fungicidal activity [25]. Polyene resistance is infrequent but has been reported to derive from sequestration of ergosterol [6,19]. The nucleoside analog 5-flucytosine (5FC), whose import into fungal cells is governed by Fcy2 *(Candida)* or FcyB *(Aspergillus),* is converted to 5-fluorouridine upon uptake into fungal cells and acts to disrupt RNA and protein synthesis because it becomes incorporated in the place of uracil [26]. Currently there are several novel antifungal agents under clinical investigation that have novel mechanisms of action, including novamycin, an antifungal peptide that interacts with the plasma membrane causing cell lysis [6], olorofim [27,28] of the orotomide class of drugs that inhibit pyrimidine biosynthesis via reversible inhibition of mitochondrial dihydroorotate dehydrogenase, and fosmanogepix, a glycosylphosphatidylinositol (GPI) inhibitor that inhibits the activity of Gwt1 to prevent GPI anchoring [29,30].

There are multiple bottlenecks in the successful development of antifungal drugs, such as the development of high-throughput screens, maximizing target specificity and bioavailability, and minimizing toxicity in the host. Although *in silico* approaches have the potential to accelerate initial stages of drug development, their success is dependent upon high-resolution protein structures, as well as upon robust mechanistic understanding of the signaling pathways and the effects of therapeutic intervention on fungal physiology.

## Lessons from drug discovery in humans

Integral membrane proteins such as GPCRs, ion channels, and transporters are among the protein families that are most targeted by small-molecule therapeutics in humans [31,32]. These complex membrane proteins play important physiological roles and their location on the cell surface makes them readily accessible to pharmacological agents. Membrane proteins account for >60% of FDA-approved drugs, and GPCRs alone are targets for 34% of all clinical drugs, followed by ion channels which are targets for 19% of all drug agents [33,34]. GPCRs are ideal drug targets because they are master switches for a variety of physiologically important signal transduction cascades. There are 475 marketed drugs that target 108 receptors in humans that belong to the GPCR superfamily [33,35], and many more are in clinical trials [36].

The development of a new drug from conception to an approved product in the market is a complex process which can take in excess of 10 years and costs an estimated $1.1 billion in R&D costs [37]. The traditional drug discovery process involves screening of large libraries of compounds in a high-throughput fashion against the drug target or using a cell-based assay with a readout that is dependent on the activity of the drug target [38]. Despite automation, biochemical analysis of hundreds of thousands of compounds by high-throughput screening involves significant costs and time [39]. *In silico* high-throughput screening of structures offers a cheaper and powerful alternative to identify novel small-molecule hits from large libraries of virtual compounds, and is gradually becoming the next-generation drug discovery approach of choice [36,40]. There is thus considerable interest in determining new structures of GPCRs, transporters, and ion channels, particularly now that single-particle cryo-EM has made it easier to determine structures compared to X-ray crystallography [41]. Once structures have been determined, ultra-large *in silico* libraries and computational platforms can screen >11 billion compounds in a matter of months and have been effective at identifying multiple hits that have nanomolar affinity for GPCRs [13,14]. A key prerequisite to structure determination of a fungal membrane protein to eventually develop antifungal agents is an understanding of its role in virulence and pathogenicity. Target validation is crucial in the drug discovery process and, in the context of antifungals, asks the basic question ‘will inhibiting or activating this protein kill or prevent the growth of the fungus, lessen its virulence, or reduce adverse immunopathology?’ The availability of genetically tractable model organisms such as *Saccharomyces cerevisiae* has promoted a thorough understanding of the biological processes involving GPCRs, ion channels, and transporters, many of which are conserved in pathogenic yeasts and fungi where they are indispensable for virulence or viability (Table 1). There are therefore many exciting opportunities for the development of antifungal drugs directed to targets in the plasma membrane.

## Potentially druggable GPCRs in fungal pathogens

**Figure 2:**
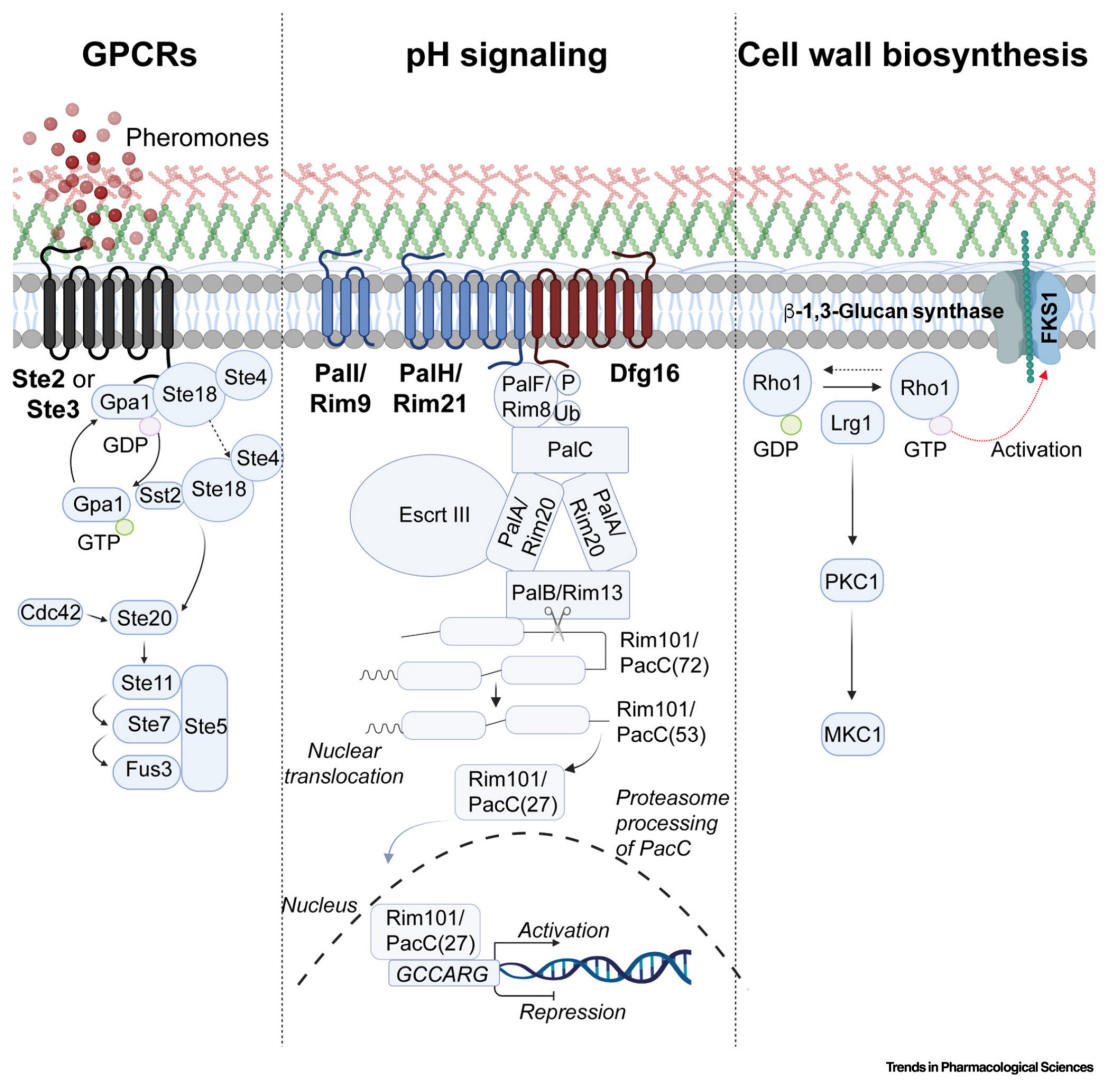
Signaling pathways of conserved fungal plasma membrane proteins required for virulence. Activation of Ste2, a conserved pheromone-sensing class D GPCR (G protein-coupled receptor), results in stimulation of MAPK signaling and cell cycle arrest. The pH-sensing mechanism is highly conserved across many fungal pathogens and governs activation of the pH-responsive transcription factor Rim101/PacC which regulates adaptation to alkaline environments. The functionality of the pH-sensing mechanism also influences fungal susceptibility to various antifungals including echinocandins and 5-flucytosine (5FC). Rho1, a GTPase, is essential in several fungal pathogens and regulates cellular morphogenesis via mechanisms such as the activation of β-1,3-glucan synthase, and is a crucial component of the cell wall integrity pathway. Image created using BioRender.

In mammalian hosts, invasive fungal growth requires appropriate coupling between sensory perception and signal transduction to enable adaptation to the host environment and evasion of immune defenses. Although crucial for fungal pathogenicity and immune evasion, virulence factors have not been prioritized for drug discovery, which has traditionally focused upon the essential physiology of the fungal cell. Fungus-specific GPCRs, ion channels, and transporters located in the plasma membrane are often required for virulence and/or viability and, in light of the tractability and throughput of newer technologies, could become attractive targets for novel antifungal drug discovery (Table 1 and Figure 2). It is feasible that selective impairment of pathogenic traits would provide a means to disarm fungal pathogens without exerting selective pressure to evolve drugresistance mechanisms. In harmony with residual host innate immune responses, this approach might deliver sufficient moderation of virulence to obviate the need for toxic antifungal drugs [42]. For example, in *Candida* spp., the GPCR GPR1 acts as a biosensor for extracellular lactate, resulting in activation of PKA, regulation of the yeast to hypha switch that is essential for tissue invasion, expression of the pore-forming toxin candidalysin (Ece1p) that is essential for cytolysis of human cells, and Crz1-mediated **β-glucan masking** that promotes immune evasion [43]. Mutant strains lacking both Gpr1 and the G protein α-subunit Gpa2 exhibit a complete loss of β-glucan masking, whereas those lacking only the receptor exhibit attenuated, but not ablated, masking [43].

*Cryptococcus neoformans* is another pathogenic fungus that uses GPCRs in several virulence mechanisms, in particular the morphological transition to giant or Titan cells, (**Titanization**) [44,45]. This involves several GPCRs including Ste3 (the a-pheromone receptor), GPR4 (activates cAMP/PKA following sensing of amino acids), and GPR5 (activates cAMP/PKA, ligand unknown) [46,47]. Mice infected with a *gpr4Δ gpr5Δ* double-mutant strain of *C. neoformans* showed enhanced survival compared to mice infected with wild-type (WT) *C. neoformans*. There was 100% mortality in mice infected with WT pathogen after 21-27 days, whereas only 40% of the mice infected with the *gpr4Δ gpr5Δ* mutant succumbed to infection by 61 days post-infection [44,45].

Fifteen GPCRs have been identified in the major mold pathogen of humans, *Aspergillus fumigatus,* some of which are not conserved in yeasts but are essential for virulence in filamentous fungi [48]. For example, the GPCR GprK (activated by sensing of pentose sugars) [49] has many roles, including in asexual development, carbon sensing, stress responses, and the production of gliotoxin, the most potent toxin produced by *A. fumigatus* [50]. The *gprK* null mutant exhibited a 47% reduction in invasiveness comparative to the WT in the human epithelial cell line A549. The cell-wall integrity pathway is crucial for fungal viability, and the GPCR GprM, which is activated by glucose, is involved in its regulation via phosphorylation of **mitogen-activated protein kinase (MAPK)**, MpkA [51], as well as in mycotoxin production and secondary metabolism. In a *Galleria mellonella* (wax moth larva) model of infection, the *ΔgprM* mutant of *A. fumigatus* exhibited a 60% reduction in mortality relative to WT and reconstituted counterparts [51].

## Plasma membrane sensors of pH in virulence

pH adaptation is another crucial determinant of virulence in most fungal pathogens of humans, plants, animals, and insects that is a potential target for drug development. Pathogenic fungi have evolved to adapt to and commandeer external pH to their benefit, making perturbation of pH signaling a highly attractive strategy for novel antifungal drug discovery [52]. pH adaptation is driven by the fungus-specific **Rim101/PacC signaling pathway** that involves a transcription factor whose nuclear localization is governed by pH-dependent proteolytic processing [53].

Rim101/PacC signaling regulates a wide range of cellular and biological processes that are essential for pathogenicity, including adhesion to epithelia, biofilm formation, the yeast to hypha switch, morphogenesis, and nutrient transport and metabolism; mice infected with pH signaling-defective mutants of *Candida* most often survive [54]. Activation of the Rim101/PacC pathway requires the sensing of ambient pH by an integral membrane protein, Rim21/PalH, that contains seven transmembrane regions. In *Candida* species, the functionality of Rim21 requires a cognate arrestin, Rim8, and two further membrane proteins Rim9 and Dfg16 [55,56]. In *Candida* species, Rim101 signaling mediates tolerance to echinocandins (micafungin and anidulafungin) through activation of the essential **chaperone** Hsp90 and inositol phosphoryl transferase IPT1, an enzyme which catalyzes the synthesis of sphingolipid mannose-(inositol-P)_2_-ceramide [57]. Mutants defective for Rim101 signaling also exhibit hypersensitivity to azoles (fluconazole, voriconazole, and posaconazole) [57]. PacC null strains of *A. fumigatus* also exhibit increased sensitivity to antifungals [53]; moreover, PacC-mediated repression of the importer FcyB promotes insensitivity to the antifungal 5FC [26]. Consequently, 5FC is not used clinically to treat invasive *Aspergillus* infections [26]. Therefore, in the case of infections caused by *Candida* and *Aspergillus* species, combination therapies involving existing antifungal drugs and Rim101/PacC perturbation might overcome the problem of drug resistance. The propensity for fungal GPCR inhibition to moderate antifungal resistance remains unknown.

## Drug targets in cell wall biology and membrane potential generation

The integrity of the cell wall is central to fungal viability, and proteins that are essential for its maintenance are therefore potential drug targets. For example, the GTPase Rho1 in *Candida*, *Cryptococcus,* and *Aspergillus* spp. is essential for cell wall integrity and hence fungal viability [58–61]. Rho1 proteins are required for the regulation and synthesis of cell wall components through activation of MAPK signaling [62]. Rho1 acts a positive regulator of β-1,3-glucan synthase, thereby promoting β-1,3-glucan synthesis and cell wall biogenesis, as well as the organization of the actin cytoskeleton [63]. In *Candida* spp., haploinsufficiency screening revealed that depletion of Rho1 results in increased susceptibility to the antifungal agents caspofungin and calcofluor white [59,64]. Activation of MAPK and cAMP by Ras orthologs is crucial for virulence via regulation of the yeast to hypha switch, biofilm formation, and programmed cell death [65]. In *Cryptococcus* and *Saccharomyces* spp., *ras1* null mutants exhibit a growth defect at 37°C that is associated with dysregulation of actin polarization [66].

A final interesting example of an integral membrane protein that is a potential drug target is the H^+^-ATPase, Pma1. This proton pump is an essential protein that is highly conserved in fungi and generates the potential across the plasma membrane that drives nutrient import via H^+^ symport [67]. Regulation of neutral-alkaline cytosolic pH by Pma1 is conserved in several fungal pathogens. In *Candida* spp., a 90% reduction of Pma1 at the cell surface through a C-terminal truncation reduces ATPase activity by 75% and results in dysregulated glucose metabolism and disrupted hyphal formation [68]. The stability of Pma1 in *Cryptococcus* is mediated by inositol phosphosphingolipid phospholipase that promotes oligomerization [69]. Omeprazole was the first reported inhibitor of Pma1 activity [70], whereas more recently NSC11668 was identified as an inhibitor of S. *cerevisiae* Pma1 through direct competition for ATP binding [71,72].

## A recent success story: cryo-EM structure determination of the yeast GPCR Ste2

The determination of the first high-resolution structures of **Ste2**, the prototypical class D GPCR from *S. cerevisiae*, provides a relevant example for how SBDD could now be used to develop novel agents targeting fungal GPCRs [73,74]. GPCRs constitute the single largest family of membrane proteins in fungi that are crucial for their survival and reproduction [48,75]. Ste2 is a widely conserved receptor which is essential for sexual mating and senses the pheromone **α-factor** in a cell type-specific manner in yeast [76]. Activation of Ste2 ultimately results in activation of MAPK cascades and is essential for cell-cycle arrest and fusion of MATa cells with MATα cells. Ste2 was the first ligand-binding GPCR to be sequenced [77], and the genetic tractability of S. *cerevisiae* has allowed extensive characterization of the α-factor pheromone-induced, Ste2-mediated signal transduction cascade which led to many paradigms for GPCR and G protein-mediated signaling and regulation [76,78,79]. Homologous proteins in multiple human fungal pathogens (Table 1) are essential for virulence. Despite the wealth of genetic and biochemical data available from the study of Ste2 over the past three decades, the molecular and mechanistic details underlying Ste2 activation were unknown. Furthermore, the lack of structural information for Ste2 and other fungal GPCRs has hampered the development of novel antifungal agents targeting them.

**Figure 3:**
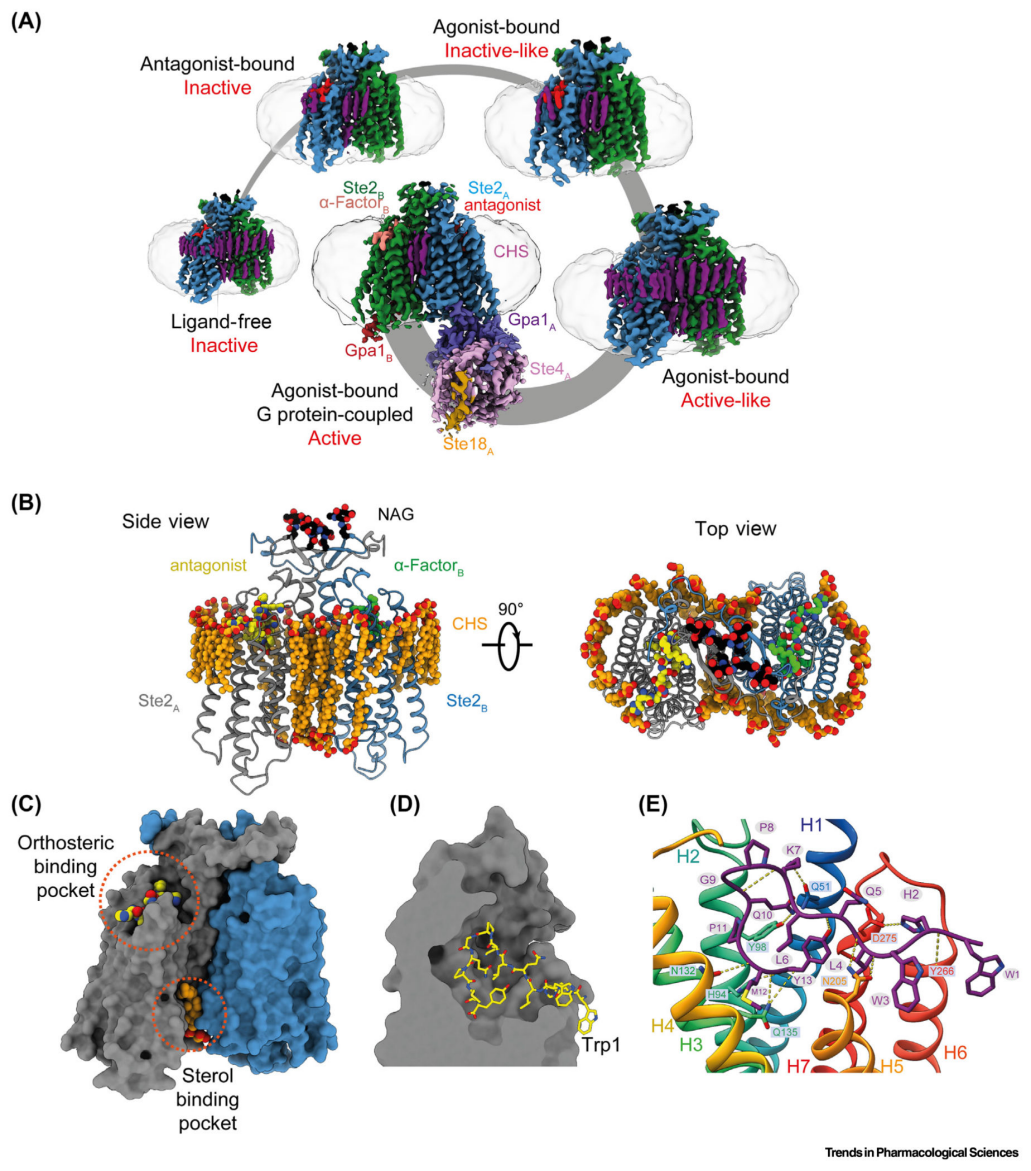
Cryo-electron microscopy (Cryo-EM) structures of Ste2 in different conformational states. (A) Structures of Ste2 in the ligand-free state, antagonistbound state, two agonist-bound intermediate states, and G protein-coupled are depicted. The detergent micelle is shown in pale gray and subunits are colored according to the key shown for the G protein-coupled state. One G protein was sufficiently well ordered to allow building of an atomic model, whereas the other G protein was mobile except for the C-terminal α-helix depicted. Subscripts denote the protomer associated with the various subunits. (B) Structural model of Ste2 in the antagonist-bound state. (Left panel) View parallel to the membrane plane (side view). (Right panel) View perpendicular to the membrane plane from the extracellular region (top view). Ste2_A_ (gray), Ste2_B_ (blue), cholesterol hemisuccinate (CHS, orange), *N*-acetylglucosamine molecules (NAG, black). Antagonist is colored according to the protomer to which it is bound (yellow or green). (C) Surface model of Ste2 in the antagonist-bound state. The orthosteric binding site and sterol-binding pocket are highlighted, where α-factor and CHS bind, respectively. (D) Cross-section of the orthosteric binding pocket in G protein-coupled Ste2 revealing the extended binding mode of α-factor in which the N-terminal Trp1 projects outside the binding pocket. (E) Atomic interactions between α-factor (purple) and Ste2 in the G protein-coupled state (rainbow coloration). Residues of α-factor and Ste2 involved in hydrogen-bonding interactions (yellow broken lines) are shown as sticks (oxygen, red; nitrogen, blue; sulfur, yellow). Reproduced, with permission, from Velazhahan *et al.* [73].

Cryo-EM was used to determine five different high-resolution structures of Ste2, including a ligand-free state, an antagonist-bound state, two agonist-bound intermediate states, and an agonist-bound G protein-coupled state (Figure 3) [73,74]. The structures led to many striking observations that could not be inferred in the absence of structural data. The structures revealed that Ste2 exists as a homodimer in all the different states in which an extensive dimer interface is formed by the domain-swapped N terminus, extracellular loop 1, and helix H1 (Figure 3A-C). The region immediately C-terminal to helix H7 in the inactive state transitions into an ordered α- helix upon Ste2 activation and contributes to the dimer interface only in the active states. The five structures of Ste2 represent a series of snapshots of Ste2 along its activation pathway and revealed a new activation mechanism that is different from all other GPCRs studied thus far. The intracellular end of helix H7 forms a random coil that sterically blocks the G protein-coupling site in the inactive states. Upon agonist binding, there is a 6 Å outward movement of the extracel-lular end of H6, followed by a 20 Å outward movement of the intracellular end of H7 that unblocks the G protein-coupling site. A12 Å inward movement of the intracellular end of H6 also occurs to enable interactions with the G protein. This activation mechanism is distinct from the prototypical mammalian class A and class B GPCRs where the primary block to G protein coupling is the cytoplasmic end of H6, which moves outwards from the receptor core by 10–15 Å upon activation to allow binding of the G protein. The G protein couples to Ste2 at the same site as observed in mammalian class A and class B GPCRs, but its orientation in relation to the receptor is different from human G proteins, and this allows two G protein heterotrimers to simultaneously couple to the Ste2 dimer. The observed structural and mechanistic differences highlight the possibility of designing drugs that are specific for fungal GPCRs without inadvertently targeting human GPCRs.

## Targeting Ste2 for drug development

The α-factor pheromone binds in an extended mode in which all 13 amino acids of the peptide make extensive interactions with the extracellular segments of all the seven transmembrane helices of the receptor and the extracellular loops (Figure 3E) [73,74]. The peptide binds in a deep cleft, with the N-terminal Trp1 projecting outside the orthosteric binding site (Figure 3D). A 6 Å outward movement of the extracellular end of H6 is necessary to accommodate this conformation of Trp1, and this structural change is crucial for receptor activation. Deletion of Trp1 of α-factor and mutation of Trp3 to alanine converts the agonist into an antagonist [80]. This structural knowledge could inform rational drug discovery. For instance, small-molecule drugs are preferred over peptides or antibodies owing to their greater *in vivo* stability, oral bioavailability, water solubility, and cell permeability [81,82]. An agonist for Ste2 should possess the ability to both activate the receptor by driving the outward movement of the extracellular end of H6 and engaging with extracellular segments throughout Ste2, and it will likely be challenging for a small molecule agonist to achieve all this. A peptide or a biologic, such as a nanobody or antibody, may be more suitable to drive the full range of structural changes at the extensive orthosteric binding pocket.

An interesting possibility raised by the structures is to target other putative binding sites in Ste2 that could bind small molecules to achieve desired pharmacological outcomes. Many sterols are visualized in the Ste2 structure (Figure 3B), and some are interesting because they are present in the inactive state structures and not in the active state structures, suggesting that they could be exploited for the design of small-molecule antagonists. For example, a sterol binds at an intracellular cleft in the inactive states of Ste2, and sterically prevents H7 from unblocking the G protein coupling site (Figure 3C). The sterol is modeled as cholesterol hemisuccinate (CHS) because this was present in vast molar excess during receptor purification. Many allosteric sites in GPCRs have previously been characterized and successfully targeted to achieve diverse functional responses [83,84]. Another consideration is to determine whether an agonist or antagonist for Ste2 is desired. An antagonist that inactivates Ste2 would halt pheromone-induced sexual reproduction; abrogating the function of Ste2-like receptors has been demonstrated to decrease virulence and antifungal resistance development in candidiasis and pulmonary aspergillosis [85–88]. By contrast, an agonist to Ste2 would arrest cell growth in the G1 phase of the cell cycle and could be utilized in combination with other antifungal regimens [89]. The availability of structural data has allowed the identification of novel binding sites, which would otherwise remain unknown, and guides drug development strategies.

## Concluding remarks

The structures of fungal GPCRs had remained enigmatic despite the efforts of multiple groups over the past two decades because of the lack of structural tools to stabilize and study these receptors. We have developed new tools, such as fungal mini-G proteins, to stabilize the active G protein-coupled state and ‘pre-stabilization of a GPCR by weak association’ (PSGWAY) methodology to stabilize ligand-free states of GPCRs for structural studies [73,74]. These structural tools could now be easily adapted to determine the structures of other fungal GPCRs, including those from pathogenic species (see Outstanding questions). Our work demonstrates the tractability of using the baculovirus overexpression system for the production of fungal GPCRs in large quantities in insect cells, and which are then purified for structure determination. Membrane protein overexpression in insect cells has several advantages such as high yields, relative ease of setting up large-scale cultures, and the ability to perform most post-translational modifications [90,91]. It also helps to circumvent challenges presented by membrane protein overexpression in yeast, such as the requirement for harsh lysis procedures to disrupt the cell wall, as well as the activation of native signaling pathways that leads to poor expression levels and reduced cell growth.

Advances in cryo-EM structure determination methods have resulted in an incredible increase in the speed at which novel membrane protein structures can be determined because well-diffracting crystals are unnecessary and much smaller quantities of purified proteins are required [12]. Another key advantage of cryo-EM over X-ray crystallography is that no extensive engineering of the receptor is required, such as truncation of flexible regions that may prevent crystal formation [41]. This can provide unexpected biological insights, such as the domain-swapped dimer interface formed by the N terminus of Ste2 (Figure 3B) [73,74]. Although state-of-the-art software such as AlphaFold [92] could be used to predict the structures of GPCRs, in several cases the predicted models and experimental structures were found to show differences in the shape and conformation of the ligand-binding pockets and transducer-binding interfaces, which hampers the use of the predicted models for SBDD [93]. Nevertheless, structure-prediction algorithms are expected to improve with greater availability of representative experimental structures, and could be used in tandem to gain complementary insights during drug development. Recent advances in cryo-EM allow relatively rapid determination of multiple structures of the same receptor with different ligands and/or tool compounds, which could facilitate the development of drugs with improved efficacy and potency [94]. The structural studies on Ste2 in all relevant states along its activation cycle have provided high-resolution templates for computational drug design, and these are already being exploited by other groups such as Bai *et al.* [95], and demonstrate the potential of using cryo-EM to discover new biological knowledge with implications for drug development.

## Figures and Tables

**Table 1:** Conserved plasma membrane proteins required for fungal viability or virulence

Category	Protein	*Candida*	*Cryptococcus*	*Histoplpsma*	*Pneumocystis*	*Aspergillus*	Transmembrane domains	Function	Refs
GPCR	Ste2	▴	▴	○	○	○	7	Pheromone α-factor receptor	[77,89]
	Ste3	▴	▴	○	○	○	7	Pheromone a-factor receptor	[77,89]
	GPR1	▴	▴	▴	▴	▴	7	Activates cAMP/PKA	[43]
	GPR4	▴	▴	▴	▴	▴	7	Activates cAMP/PKA	[44]
	GprK	–	–	–	–	▴	7	Regulates stress responses	[49]
	GprM	–	–	–	–	▴	7	Regulates cell wall integrity	[51]
Signal transduction	PalH/Rim21	▴	–	▴	▴	▴	7	Senses and transduces extracellular alkaline pH	[54,56]
	Dfg16	▴	–	–	–	–	7	Mutual dependency with Rim21	[54,56]
	Ras1/A	•	▴	▴	▴	▴	Lipidated	GTPase, regulates cAMP and MAPK	[66]
Cell wall synthesis	Rho1/A	•	•	▴	▴	•	Lipidated	Activates 1,3-β-glucan synthase	[60,61,63]
	1,3-β-Glucan synthase	•	•	▴	▴	•	FKS1 subunit: 14 FKS2 subunit: 16	Synthesizes 1,3-β-glucan	[21,22,96]
ATPase	Pma1/A	•	▴	▴	▴	•	10	H^+^-ATPase	[67]

Key: ○ conserved; ▴ essential for virulence; • essential for viability; - no data.

## Highlights

Integral membrane proteins found in the plasma membrane of fungi have great potential for the development of novel antifungal agents.

In-depth studies on fungal signaling pathways define proteins that when deleted result in decreased virulence or viability, highlighting their potential as drug targets.

New tools and methodologies have been developed to determine the structure of the yeast pheromone-sensing G protein-coupled receptor (GPCR) Ste2, highlighting the tractability of fungal GPCRs for structure determination.

Single-particle electron cryo-microscopy was used to determine five structures of Ste2 that show the molecular details of ligand-induced receptor activation and highlight possible receptor regions that are amenable for drug development.

## Outstanding questions

What fungal membrane proteins should be prioritized for the future development of antifungal drugs?

Can fungal membrane proteins be targeted to offset resistance to currently available antifungal drugs?

Can drugs be developed to highly conserved or essential regions to reduce the probability of drug resistance?

Should multiple drugs be given simultaneously to target different proteins so as to increase efficacy and reduce the development of resistance?

Should cryo-EM structure determination of all novel fungal drug targets be fast-tracked to combat attrition during clinical trials?

How tractable will these structures be to drug development?

## Glossary

**Chaperone:** a class of proteins that promote conformational protein folding, block aggregation, disaggregate proteins, and assist in protein degradation.

**Echinocandins:** a class of antifungal drugs that inhibit the enzyme β-1,3-D-glucan synthase and prevent synthesis of β-1,3-glucan which is necessary for the integrity of the fungal cell wall.

**Electron cryo-microscopy (cryo-EM):** a structural biology method used to determine the 3D structures of macromolecules. The technique involves flash-freezing of biological molecules, such as proteins, in solution, and then bombarding them with electrons to obtain images of individual molecules which are then used to reconstruct the 3D structure of the molecule.

**α-Factor:** a 13 amino acid pheromone synthesized by haploid α cells of *Saccharomyces cerevisiae* which binds to Ste2, a GPCR present in haploid a cells, and mediates sexual mating.

**FKS1 subunit:** the catalytic subunit of the enzyme β-1,3-D-glucan synthase which directly mediates the production of the major and essential cell-wall component β-1,3-glucan.

**β-Glucan masking:** β-1,3-glucan in fungal cell walls is a highly immunogenic epitope that is recognized by host immune receptors leading to an antifungal immune response. β-Glucan masking refers to the phenomenon where the β-1,3-glucan is covered (masked) by a layer of glycosylated proteins on the outer surface of the fungal cell wall, thereby hiding it from host immune detection.

**G protein-coupled receptors (GPCRs):** integral membrane proteins that contain seven transmembrane helices and convert extracellular signals into intracellular responses.

**Mitogen-activated protein kinase (MAPK):** a highly conserved signaling cascade that regulates cell proliferation, differentiation, and death.

**Protein structure:** the 3D arrangement of atoms in an amino acid chain molecule.

**Rim101/PacC signaling pathway:** Rim101/PacC is a transcription factor that is activated downstream of extracellular pH sensing by a specific fungal pathway, and signaling mediated by Rim101/PacC regulates various cellular processes that are essential for pathogenicity, such as adhesion to epithelia, biofilm formation, morphogenesis, nutrient transport, and metabolism.

**Ste2:** a GPCR present in haploid a cells of *Saccharomyces cerevisiae* that recognizes and binds to α-factor from haploid α cells and leads to an intracellular signaling response that is necessary for sexual mating.

**Structure-based drug discovery (SBDD):** the design and optimization of chemical structures ofdrug compounds based on the structure of the biological target.

**Titanization:** a process that leads to formation of infectious titan cells of *Cryptococcus* which are enlarged cells of 50-100 μm in diameter and that exhibit several features that modulate their interactions with the host immune system.

**Triterpenoids:** chemical compounds containing a C30 hydrocarbon skeleton composed of six isoprene units.

## References

[1] Woolhouse M, Farrar J (2014). Policy: an intergovernmental panel on antimicrobial resistance. Nature.

[2] O’Neill J (2016). Tackling Drug-Resistant Infections Globally: Final Report and Recommendations, UK Government.

[3] Brown GD (2012). Hidden killers: human fungal infections. Sci Transl Med.

[4] World Health Organization (2022). WHO Fungal Priority Pathogens List to Guide Research, Development and Public Health Action.

[5] Miller RA (2018). A case for antifungal stewardship. Curr Fung Infect Rep.

[6] Hoenigl M (2021). The antifungal pipeline: fosmanogepix, ibrexafungerp, olorofim, opelconazole, and rezafungin. Drugs.

[7] Jenks JD (2018). Spotlight on isavuconazole in the treatment of invasive aspergillosis and mucormycosis: design, development, and place in therapy. Drug Des Devel Ther.

[8] Fisher MC (2022). Tackling the emerging threat of antifungal resistance to human health. Nat Rev Microbiol.

[9] De Nobel J, Barnett J (1991). Passage of molecules through yeast cell walls: a brief essay-review. Yeast.

[10] Lyumkis D (2019). Challenges and opportunities in cryo-EM single-particle analysis. J Biol Chem.

[11] Kühlbrandt W (2014). The resolution revolution. Science.

[12] Danev R (2021). Routine sub-2.5 Å cryo-EM structure determination of GPCRs. Nat Commun.

[13] Lyu J (2019). Ultra-large library docking for discovering new chemotypes. Nature.

[14] Sadybekov AA (2022). Synthon-based ligand discovery in virtual libraries of over 11 billion compounds. Nature.

[15] Keighley C (2021). Consensus guidelines for the diagnosis and management of invasive candidiasis in haematology, oncology and intensive care settings, 2021. Intern. Med J.

[16] Aoyama Y (1984). Yeast cytochrome P-450 catalyzing lanosterol 14 alpha-demethylation. II. Lanosterol metabolism by purified P-450(14)DM and by intact microsomes. J Biol Chem.

[17] Georgopapadakou NH, Walsh TJ (1996). Antifungal agents: chemotherapeutic targets and immunologic strategies. Antimicrob Agents Chemother.

[18] Ghannoum MA, Rice LB (1999). Antifungal agents: mode of action, mechanisms of resistance, and correlation of these mechanisms with bacterial resistance. Clin Microbiol Rev.

[19] Arastehfar A (2021). Aspergillus fumigatus and aspergillosis: from basics to clinics. Stud Mycol.

[20] Arastehfar A (2020). Drug-resistant fungi: an emerging challenge threatening our limited antifungal armamentarium. Antibiotics.

[21] Jallow S, Govender NP (2021). Ibrexafungerp: a first-in-class oral triterpenoid glucan synthase inhibitor. J Fungi.

[22] Pinto e Silva A (2020). FKS1 mutation associated with decreased echinocandin susceptibility of Aspergillus fumigatus following anidulafungin exposure. Sci Rep.

[23] Jiménez-Ortigosa C (2017). Emergence of echinocandin resistance due to a point mutation in the fks1 gene of Aspergillus fumigatus in a patient with chronic pulmonary aspergillosis. Antimicrob Agents Chemother.

[24] Siopi M (2022). Pan-echinocandin resistant C. parapsilosis harboring an F652S Fks1 alteration in a patient with prolonged echinocandin therapy. J Fungi.

[25] Stone NR (2016). Liposomal amphotericin B (AmBisome®): a review of the pharmacokinetics, pharmacodynamics, clinical experience and future directions. Drugs.

[26] Gsaller F (2018). Mechanistic basis of pH-dependent 5-flucytosine resistance in Aspergillus fumigatus. Antimicrob Agents Chemother.

[27] Oliver JD (2016). F901318 represents a novel class of antifungal drug that inhibits dihydroorotate dehydrogenase. Proc Natl Acad Sci.

[28] du Pré S (2018). Effect of the novel antifungal drug F901318 (olorofim) on growth and viability of Aspergillus fumigatus. Antimicrob Agents Chemother.

[29] Tsukahara K (2003). Medicinal genetics approach towards identifying the molecular target of a novel inhibitor of fungal cell wall assembly. Mol Microbiol.

[30] Miyazaki M (2011). In vitro activity of E1210 a novel antifungal, against clinically important yeasts and molds. Antimicrob Agents Chemother.

[31] Sriram K, Insel PA (2018). G protein-coupled receptors as targets for approved drugs: how many targets and how many drugs? Mol. Pharmacol.

[32] Robertson MJ (2022). Drug discovery in the era of cryoelectron microscopy. Trends Biochem Sci.

[33] Hauser AS (2017). Trends in GPCR drug discovery: new agents, targets and indications. Nat Rev Drug Discov.

[34] Charlton FW (2020). Ion channels as therapeutic targets for viral infections: further discoveries and future perspectives. Viruses.

[35] Hauser AS (2018). Pharmacogenomics of GPCR drug targets. Cell.

[36] Congreve M (2020). Impact of GPCR structures on drug discovery. Cell.

[37] Wouters OJ (2020). Estimated research and development investment needed to bring a new medicine to market, 2009-201 8. Jama.

[38] Hughes JP (2011). Principles of early drug discovery. Br J Pharmacol.

[39] Lin X (2020). A review on applications of computational methods in drug screening and design. Molecules.

[40] Liu L, Jockers R (2020). Structure-based virtual screening accelerates GPCR drug discovery. Trends Pharmacol Sci.

[41] García-Nafría J, Tate CG (2021). Structure determination of GPCRs: cryo-EM compared with X-ray crystallography. Biochem Soc Trans.

[42] Vu K (2019). Cryptococcal meningitis and anti-virulence therapeutic strategies. Front Microbiol.

[43] Ballou ER (2016). Lactate signalling regulates fungal β-glucan masking and immune evasion. Nat Microbiol.

[44] Crabtree JN (2012). Titan cell production enhances the viru-lenceof Cryptococcus neoformans. Infect Immun.

[45] Okagaki LH (2011). Cryptococcal titan cell formation is regulated by G-protein signaling in response to multiple stimuli. Eukaryot Cell.

[46] Zaragoza O (2019). Basic principles of the virulence of Cryptococcus. Virulence.

[47] Yang C (2022). Cryptococcus escapes host immunity: what do we know? Front. Cell Infect Microbiol.

[48] Brown NA (2018). Fungal G-protein-coupled receptors: mediators of pathogenesis and targets for disease control. Nat Microbiol.

[49] Jung MG (2016). Characterization of gprK encoding a putative hybrid G-protein-coupled receptor in Aspergillus fumigatus. PLoSOne.

[50] Knowles SL (2020). Gliotoxin, a known virulence factor in the major human pathogen Aspergillus fumigatus, is also biosynthesized by its nonpathogenic relative Aspergillus fischeri. mBio.

[51] Filho A (2020). Aspergillus fumigatus G-protein coupled receptors GprM and GprJ are important for the regulation of the cell wall integrity pathway, secondary metabolite production, and virulence. mBio.

[52] Sherrington SL (2018). Host sensing by pathogenic fungi. Adv Appl Microbiol.

[53] Bertuzzi M (2014). The pH-responsive PacC transcription factor of Aspergillus fumigatus governs epithelial entry and tissue invasion during pulmonary aspergillosis. PLoS Pathog.

[54] Davis D (2000). Candida albicans RIM101 pH response pathway is required for host-pathogen interactions. Infect Immun.

[55] Gomez-Raja J, Davis DA (2012). The β-arrestin-like protein Rim8 is hyperphosphorylated and complexes with Rim21 and Rim101 to promote adaptation to neutral-alkaline pH. Eukaryot Cell.

[56] Barwell KJ (2005). Relationship of DFG16 to the Rim101p pH response pathway in Saccharomyces cerevisiae and Candida albicans. Eukaryot Cell.

[57] Garnaud C (2018). The Rim pathway mediates antifungal tolerance in Candida albicans through newly identified Rim101 transcriptional targets, including Hsp90 and Ipt1. Antimicrob. Agents Chemother.

[58] Salvatori O (2018). Candida albicans Ras1 inactivation increases resistance to phagosomal killing by human neutrophils. Infect Immun.

[59] Smith SE (2002). Candida albicans RHO1 is required for cell viability in vitro and in vivo. FEMS Yeast Res.

[60] Lam WC (2013). Role of Cryptococcus neoformans Rho1 GTPases in the PKC1 signaling pathway in response to thermal stress. Eukaryot Cell.

[61] Guest GM (2004). Aspergillus nidulans RhoA is involved in polar growth, branching, and cell wall synthesis. Fungal Genet Biol.

[62] Dichtl K (2016). Cell wall integrity signalling in human pathogenic fungi. Cell Microbiol.

[63] Qadota H (1996). Identification of yeast Rho1p GTPase as a regulatory subunit of 1, 3-β-glucan synthase. Science.

[64] Becker JM (2010). Pathway analysis of Candida albicans survival and virulence determinants in a murine infection model. Proc Natl Acad Sci.

[65] Phillips AJ (2006). Ras pathway signaling accelerates programmed cell death in the pathogenic fungus Candida albicans. Proc Natl Acad Sci.

[66] Alspaugh JA (2000). RAS1 regulates filamentation, mating and growth at high temperature of Cryptococcus neoformans. Mol Microbiol.

[67] Gong X, Chang A (2001). A mutant plasma membrane ATPase, Pma1-10 is defective in stability at the yeast cell surface. Proc Natl Acad Sci.

[68] Rane HS (2019). Candida albicans Pma1p contributes to growth, pH homeostasis, and hyphal formation. Front Microbiol.

[69] Farnoud AM (2014). Inositol phosphosphingolipid phospholipase C1 regulates plasma membrane ATPase (Pma1) stability in Cryptococcus neoformans. FEBS Lett.

[70] Monk BC (1995). The yeast plasma membrane proton pumping ATPase is a viable antifungal target. I. Effects of the cysteine-modifying reagent omeprazole. Biochim Biophys Acta.

[71] Cohrt KAO (2018). Novel zinc-attenuating compounds as potent broad-spectrum antifungal agents with in vitro and in vivo efficacy. Antimicrob Agents Chemother.

[72] Ottilie S (2018). Two inhibitors of yeast plasma membrane ATPase 1 (ScPma1p): toward the development of novel antifungal therapies. J Cheminform.

[73] Velazhahan V (2021). Structure of the class D GPCR Ste2 dimer coupled to two G proteins. Nature.

[74] Velazhahan V (2022). Activation mechanism of the class D fungal GPCR dimer Ste2. Nature.

[75] Xue C (2008). Magnificent seven: roles of G protein-coupled receptors in extracellular sensing in fungi. FEMS Microbiol Rev.

[76] Naider F, Becker JM (2004). The α-factor mating pheromone of Saccharomyces cerevisiae: a model for studying the interaction of peptide hormones and G protein-coupled receptors. Peptides.

[77] Burkholder AC, Hartwell LH (1985). The yeast α-factor receptor: structural properties deduced from the sequence of the STE2 gene. Nucleic Acids Res.

[78] Naider F, Becker JM (2020). A paradigm for peptide hormone-GPCR analyses. Molecules.

[79] Alvaro CG, Thorner J (2016). Heterotrimeric G protein-coupled receptor signaling in yeast mating pheromone response. J Biol Chem.

[80] Raths S (1988). Peptide analogues compete with the binding of alpha-factor to its receptor in Saccharomyces cerevisiae. J Biol Chem.

[81] Wang L (2022). Therapeutic peptides: current applications and future directions. Signal Transduct Target Ther.

[82] Ngo HX, Garneau-Tsodikova S (2018). What are the drugs of the future?. MedChemComm.

[83] Lane JR (2013). Regulation of G protein-coupled receptors by allosteric ligands. ACS Chem Neurosci.

[84] Draper-Joyce CJ (2021). Positive allosteric mechanisms of adenosine A1 receptor-mediated analgesia. Nature.

[85] Seo JA (2004). The gprA and gprB genes encode putative G protein-coupled receptors required for self-fertilization in Aspergillus nidulans. Mol Microbiol.

[86] Szewczyk E, Krappmann S (2010). Conserved regulators of mating are essential for Aspergillus fumigatus cleistothecium formation. Eukaryot Cell.

[87] Ene IV, Bennett RJ (2014). The cryptic sexual strategies of human fungal pathogens. Nat Rev Microbiol.

[88] Daniels KJ (2006). Opaque cells signal white cells to form biofilms in Candida albicans. EMBO J.

[89] Alvaro CG (2014). Specific α-arrestins negatively regulate Saccharomyces cerevisiae pheromone response by downmodulating the G-protein-coupled receptor Ste2. Mol. Cell Biol.

[90] Wiseman DN (2020). Expression and purification of recombinant G protein-coupled receptors: a review. Protein Expr Purif.

[91] McKenzie EA, Abbott WM (2018). Expression of recombinant proteins in insect and mammalian cells. Methods.

[92] Jumper J (2021). Highly accurate protein structure prediction with AlphaFold. Nature.

[93] He XH (2023). AlphaFold2 versus experimental structures: evaluation on G protein-coupled receptors. Acta Pharmacol Sin.

[94] Zhang X (2021). Evolving cryo-EM structural approaches for GPCR drug discovery. Structure.

[95] Bai Q (2021). WADDAICA: a webserner for aiding protein drug design by artificial intelligence and classical algorithm. Comput Struct Biotechnol J.

[96] Castelo-Branco D (2022). Collateral consequences of agricultural fungicides on pathogenic yeasts: a One Health perspective to tackle azole resistance. Mycoses.

